# Supplementary material for: The role of contributing factors, triggers, and prodromal symptoms in the etiological classification of out-of-hospital cardiac arrest; A scoping review
Source: PLoS One. 2025 Jul 16;20(7):e0327651. doi: 10.1371/journal.pone.0327651 (PMC12266415; doi:10.1371/journal.pone.0327651)
Supplement: S2 Appendix — (DOCX) [file pone.0327651.s002.docx]

**S2 Appendix: Search strategy for conducting this scoping review**

**All Ovid Medline <1946 – 28 June 2024>**

**Search Strategy:**

1 exp *heart arrest/ep, et, sn 8584

2 OHCA.ti,ab. [Look for acronym in title and abstract] 3192

3 ("out-of-hospital" adj2 arrest).ti,ab,kf. 6920

4 ("out-of-hospital" adj2 cardiac).ti,ab,kf. 7219

5 ("out-of-hospital" adj2 heart).ti,ab,kf. 37

6 Death, Sudden, Cardiac/ep, et, sn 10428

7 (Sudden Cardiac adj2 Death).ti,ab,kf. 17577

8 or/1-7 33211

9 randomized controlled trial.pt. or randomized.mp. or randomised.mp. or placebo.mp. 1029121

10 8 not 9 30698

11 ambulance*.tw. 11491

12 emergency medical tech:.ti,ab,kf. 1250

13 EMT.ti,ab. 26287

14 first responder*.ti,ab,kf. 2702

15 paramedic.ti,ab,kf. 2880

16 exp *emergency medical services/ 99347

17 exp *emergency responders/ 9804

18 (out-of-hospital adj2 (treatment or diagnosis)).ti,ab,kf. 277

19 incident report*.ti,ab. 2324

20 witness.ti,ab. 6876

21 or/11-19 143228

22 (diagnosis or report).ti,ab,kf. or di.fs. 5226068

23 21 and 22 28129

24 (aetiology or etiology).ti,ab,kf. 301274

25 autops*.ti,ab,kf. 78187

26 ep.fs. [epidemiology as a subheading] 1878435

27 et.fs. [etiology. as a subheading] 2635211

28 exp causality/ 912940

29 cause of death/ 51948

30 (cause or causation).ti,ab,kf. 1083412

31 differential diagnosis/ 461611

32 epistry.af. 58

33 precipitated.contributing ti,ab,kf. 25626

34 presumed cause.mp. 257

35 (relative risk: or risks).tw. or cohort stud:.mp. 765592

36 or/23-35 6195716

37 exp mortality/ or mo.fs. 834257

38 exp Prognosis/ 1779927

39 prognosis.sh. or diagnosed.tw. or cohort:.mp. or predictor:.tw. or death.tw. or exp models, statistical/ 3029968

40 exp survival analysis/ 320015

41 survival.ti,ab,kf. 1045131

42 exp Treatment Outcome/ 1153843

43 37 or 38 or 39 or 40 or 41 or 42 4782420

44 10 and 36 and 43 18341

45 exp animals/ not humans.sh. 4909955

46 limit 44 to (adaptive clinical trial or address or autobiography or bibliography or biography or case reports or comment or dictionary or directory or duplicate publication or editorial or equivalence trial or evaluation study or "expression of concern" or festschrift or interactive tutorial or interview or lecture or legal case or legislation or letter or news or newspaper article or observational study, veterinary or patient education handout or periodical index or personal narrative or portrait or randomized controlled trial or randomized controlled trial, veterinary or retracted publication or "retraction of publication" or technical report or twin study or video-audio media or webcast) 3803

47 45 or 46 4913734

48 44 not 47 14139

**EBM Reviews - Cochrane Database of Systematic Reviews <2005 to June 28, 2024>**

**Search Strategy**:

--------------------------------------------------------------------------------

1 Out of hospital cardiac arrest.mp. [mp=title, short title, abstract, full text, keywords, caption text] (21)

2 OHCA.ti. (0)

3 (out-of-hospital adj3 arrest).mp. (21)

4 (out-of-hospital adj3 cardiac).ti. (5)

5 (out-of-hospital adj3 heart).ti. (0)

6 Sudden cardiac death.ti. (3)

7 1 or 2 or 3 or 4 or 5 or 6 (23)

**Embase Classic+Embase <1947 to 2024 June 28>**

**Search Strategy:**

--------------------------------------------------------------------------------

1 exp "out of hospital cardiac arrest"/ (10662)

2 OHCA.ti,ab. (5773)

3 (out-of-hospital adj3 arrest).ti,ab,kw. (11013)

4 (out-of-hospital adj3 attack).ti,ab,kw. (12)

5 (out-of-hospital adj3 heart).ti,ab,kw. (72)

6 (out-of-hospital adj3 infarction).ti,ab,kw. (89)

7 exp sudden cardiac death/ (16925)

8 heart arrest/ (76581)

9 cardiopulmonary resuscitation/ (121036)

10 (heart arrest or cardiopulmonary resuscitation or CPR).ti,ab,kw. (37900)

11 1 or 2 or 3 or 4 or 5 or 6 or 7 or 8 or 9 or 10 (198129)

12 exp ambulance/ (15693)

13 ambulance.ti,ab,kw. (15812)

14 (EMT or emergency medical tech* or paramedic or first respond*).ti,ab,kw. (44393)

15 exp *emergency medical services/ (50462)

16 exp *emergency responders/ (3939)

17 12 or 13 or 14 or 15 or 16 (111452)

18 11 and 17 (7461)

19 (aetiology or etiology).ti,ab,kw. (418072)

20 exp etiology/ (2024629)

21 exp causality/ (3220)

22 (cause or causation).ti,ab,kw. (1509780)

23 exp coroner/ (3041)

24 (coroner* or coronial or medical examiner* or death certificate*).ti,ab,kw. (17046)

25 data collection.mp. (117206)

26 ep.fs. (1104591)

27 et.fs. (2518360)

28 exp epidemiology/ (3727390)

29 epidemiology.ti,ab,kw. (292645)

30 epistry.af. (108)

31 factual database*.ti,ab,kw. (55)

32 information processing/ (246530)

33 precipitated.tw. (35504)

34 presumed cause.mp. (375)

35 prognosis.sh. or diagnosed.tw. or cohort:.mp. or predictor:.tw. or death.tw. or exp models, statistical/ [HIRU prognosis] (4052897)

36 registries/ or registr*.ti,ab,kw. (392747)

37 exp retrospective study/ (1028057)

38 survival analysis/ (26080)

39 survival.tw. (1485380)

40 or/19-39 (12185243)

41 18 and 40 (4409)

42 limit 41 to (human and english language) (3800)

43 remove duplicates from 42 (3762)
