# Supplementary material for: The role of contributing factors, triggers, and prodromal symptoms in the etiological classification of out-of-hospital cardiac arrest; A scoping review
Source: PLoS One. 2025 Jul 16;20(7):e0327651. doi: 10.1371/journal.pone.0327651 (PMC12266415; doi:10.1371/journal.pone.0327651)
Supplement: S3 Appendix — (DOCX) [file pone.0327651.s003.docx]

**S 3 Appendix: Summary of included studies evaluating the association of contributing factors with out-of-hospital cardiac arrest (OHCA) etiologies**

| **Author** | **Year/**  **Country** | **Study**  **Design** | **Source of initial**  **classification** | **Source of final diagnosis data;**  **if reported** | **Total population:**  **N** | **Initial etiologies** | **Final etiologies;**  **if reported** | **Contributing factors** |
| --- | --- | --- | --- | --- | --- | --- | --- | --- |
| **Covid-19 infection** | | | | | | | | |
| Hubert, et al ^[^[^1^](#_ENREF_1)^]^ | 2020  France | Cohort | French national OHCA registry |  | 670 | Presumed cardiac | 1-Respiratory 2-Cardiac | Covid-19 infection |
| Baert, et al ^[^[^2^](#_ENREF_2)^]^ | 2020  France | Observational | French national OHCA registry |  | 2,625 | Medical | 1-Cardiac 2-Respiratory 3-Other medical etiologies | Covid-19 infection |
| Baldi, et al ^[^[^3^](#_ENREF_3)^]^ | 2020  Italy | Observational | The Lombardia CAR |  | 811 | Medical | Medical | Covid-19 infection |
| Fothergill, et al ^[^[^4^](#_ENREF_4)^]^ | 2021  UK | Observational | LAS | Multiple sources | 4,846 | Presumed cardiac | 1-Cardiac 2-Medical  3-Trauma | Covid-19 infection |
| Sultanian, et al ^[^[^5^](#_ENREF_5)^]^ | 2021  Sweden | observational | SRCR |  | 1,946* | Medical | 1-Medical  2-Asphyxia | Covid -19 infection |
| **Antipsychotic medications** | | | | | | | | |
| Teodorescu, et al ^[^[^6^](#_ENREF_6)^]^ | 2013  USA | Observational | EMS data |  | 818 | Presumed cardiac | Not noted; toxicology not done | Antipsychotic and  Antidepressant medications |
| Allan, et al ^[^[^7^](#_ENREF_7)^]^ | 2019  Canada | Cohort | Epistry | Autopsy report  Toxicology results | 608 | OHCA of no obvious causes | Cardiac and  Other etiologies | 1-Illicit drugs 2-Psychotropic medication 3-Psychiatric disease |
| Kauppila, et al ^[^[^8^](#_ENREF_8)^]^ | 2020  Finland | Cohort study | EMS data | Autopsy and medical reports  Toxicology results | 222 | Presumed cardiac | Not noted | Antipsychotic and antidepressant medications |
| **Drug Overdose** | | | | | | | | |
| Tseng, et al ^[^[^9^](#_ENREF_9)^]^ | 2018  USA | Cohort | CARES | Autopsy report | 525 | Presumed cardiac | Cardiac and  non-cardiac etiologies | 1-Psychiatric disorder  2-Alcohol or illicit drug use |
| Rodriguez, et ^[^[^10^](#_ENREF_10)^]^ | 2021  USA | Cohort | EMS data | Autopsy reports Toxicology results | 767 | Presumed cardiac | Drug overdose | Drug overdose |
| **Seizure/Epilepsy** | | | | | | | | |
| Bardai, et al ^[^[^11^](#_ENREF_11)^]^ | 2012  Netherlands | Observational | ARREST |  | 3,853 | Presumed cardiac |  | Epilepsy and antiepileptic medication |
| Stecker, et al ^[^[^12^](#_ENREF_12)^]^ | 2013  USA | Observational | EMS data | Autopsy report | 2,417 | Presumed cardiac | 1-Normal autopsy 2-Obstructive CAD  4-Cardiomegaly | 1-Seizure  2- Antiepileptic medications |
| Lamberts, et al ^[^[^13^](#_ENREF_13)^]^ | 2015  Netherland | Observational | ARREST  OPPEC |  | 488 | Presumed cardiac | MI | QT-prolongation drugs (depolarization -blocking drugs) |
| Legriel, et al ^[^[^14^](#_ENREF_14)^]^ | 2018  France | Observational | EMS report | Medical report | 284 | CSE |  | 1-Drug poisoning  2-Acute alcohol intoxication or withdrawal  3-Vascular disease  4-Metabolic disorder  5-Undetermined |
| Eroglu, et al ^[^[^15^](#_ENREF_15)^]^ | 2021  Denmark | Observational | Danish cardiac arrest registry |  | 387,145 | Presumed cardiac |  | 1-Epilepsy  2-Anti-epileptic medication |
| **Other contributing factors related to drowning induced OHCA** | | | | | | | | |
| Youn, et al ^[^[^16^](#_ENREF_16)^]^ | 2009  South Korea | Observational | EMS data | ICU notes | 131 | Drowning |  | 1-Unknown 2-Alcohol intoxication 3-Drug intoxication 4-Traumatic injury 5-Seizure |
| Grmec, et al ^[^[^17^](#_ENREF_17)^]^ | 2009  Slovenia | Cohort | EMS data |  | 560 | 1-Presumed cardiac  2-Drowning |  | 1-Alcohol intoxication 2-Suicide |
| Dyson, et al ^[^[^18^](#_ENREF_18)^]^ | 2013  Australia | Cohort | VACAR |  | 336 | Drowning |  | 1-Trauma 2-Suicide |
| Claesson, et al ^[^[^19^](#_ENREF_19)^]^ | 2013  Sweden | Observational | SRCR  NBFM | Autopsy reports | 2,438 | Drowning |  | 1-CAD  2-Hypothermia  3-Trauma  4-Myocardial infarction  5-Other myocardial conditions  6-Epilepsy  7-Stroke  8-Hypoglycaemia |
| Reynold, et al ^[^[^20^](#_ENREF_20)^]^ | 2019  USA | Cohort | WWDR | Autopsy and  Hospital reports | 407 | Drowning |  | Illicit substances |
| Ryan, et al ^[^[^21^](#_ENREF_21)^]^ | 2023  USA | Cohort | CARES | Not reported | 1,767 | Drowning |  | 1-Cardiac disease  2-Trauma  3-Neurological disease  4-Seizure  5-Suicide  6-Intoxication  7-Other medical and non-medical conditions |
| Reizine, et al ^[^[^22^](#_ENREF_22)^]^ | 2024  France | Observational | Multicentre data registries | ICU records | 103 | Drowning |  | 1-Cardiac disease  2-Intoxication  3-Neurological disease  4-Suicide  5-Trauma |
| **Other contributing factors** | | | | | | | | |
| Schober, et al ^[^[^23^](#_ENREF_23)^]^ | 2014  Austria | Cohort | EMS data  (Utstein style) | Hospital record | 18 | Accidental hypothermia |  | 1- Intoxication 2-Trauma 3-Metabolic disorder 4-End stage disease 5-Unknown |
| Legriel, et al ^[^[^24^](#_ENREF_24)^]^ | 2018  France | Cohort | Paris-SDEC registry |  | 247 | Neurological diseases |  | 1-Neurovascular diseases 2- Poisoning 3-Traumatic brain injury  4-Seizure 5-Miscellaneous |

**ARREST**: Amsterdam resuscitation studies. **CAD**: Coronary artery disease. **CAR**: Cardiac arrest registries. **CARES**: Cardiac arrest registry to enhance survival **CSE**: Convulsive status epilepticus. **EMS:** Emergency medical services **LAS**: London ambulance service. **ICU:** Intensive care unit. **MI**: Myocardial infarction. **MVA**: Motor vehicle accident. **NBFM**: National board of forensic medicine**. OHCA:** Out- of- hospital cardiac arrest**. OPPEC**: Out- patient population-based epilepsy cohort. **PEA**: Pulseless electrical activity. **SDEC**: Sudden death expertise centre. **SRCR**; Swedish registries for cardiopulmonary resuscitation. **VACAR**: Victorian cardiac arrest registries. **WWDR**: Western Washington Drowning Registry.

*Number of OHCA cases with Covid infection was 88

**References**

1. Hubert H, Baert V, Beuscart J-B, Chazard E. Use of out-of-hospital cardiac arrest registries to assess COVID-19 home mortality. BMC medical research methodology. 2020;20(1):305.

2. Baert V, Jaeger D, Hubert H, Lascarrou J-B, Debaty G, Chouihed T, et al. Assessment of changes in cardiopulmonary resuscitation practices and outcomes on 1005 victims of out-of-hospital cardiac arrest during the COVID-19 outbreak: registry-based study. Scandinavian journal of trauma, resuscitation and emergency medicine. 2020;28(1):119.

3. Baldi E, Sechi GM, Mare C, Canevari F, Brancaglione A, Primi R, et al. COVID-19 kills at home: the close relationship between the epidemic and the increase of out-of-hospital cardiac arrests. European heart journal. 2020;41(32):3045-54.

4. Fothergill RT, Smith AL, Wrigley F, Perkins GD. Out-of-Hospital Cardiac Arrest in London during the COVID-19 pandemic. Resuscitation plus. 2021;5:100066.

5. Sultanian P, Lundgren P, Stromsoe A, Aune S, Bergstrom G, Hagberg E, et al. Cardiac arrest in COVID-19: characteristics and outcomes of in- and out-of-hospital cardiac arrest. A report from the Swedish Registry for Cardiopulmonary Resuscitation. European heart journal. 2021;42(11):1094-106.

6. Teodorescu C, Reinier K, Uy-Evanado A, Chugh H, Gunson K, Jui J, et al. Antipsychotic drugs are associated with pulseless electrical activity: the Oregon Sudden Unexpected Death Study. Heart rhythm. 2013;10(4):526-30.

7. Allan KS, Morrison LJ, Pinter A, Tu JV, Dorian P, Rescu I. Unexpected High Prevalence of Cardiovascular Disease Risk Factors and Psychiatric Disease Among Young People With Sudden Cardiac Arrest. Journal of the American Heart Association. 2019;8(2):e010330.

8. Kauppila JP, Hantula A, Pakanen L, Perkiomaki JS, Martikainen M, Huikuri HV, et al. Association of non-shockable initial rhythm and psychotropic medication in sudden cardiac arrest. International journal of cardiology Heart & vasculature. 2020;28:100518.

9. Tseng ZH, Olgin JE, Vittinghoff E, Ursell PC, Kim AS, Sporer K, et al. Prospective Countywide Surveillance and Autopsy Characterization of Sudden Cardiac Death: POST SCD Study. Circulation. 2018;137(25):2689-700.

10. Rodriguez RM, Tseng ZH, Montoy JCC, Repplinger D, Moffatt E, Addo N, et al. NAloxone CARdiac Arrest Decision Instruments (NACARDI) for targeted antidotal therapy in occult opioid overdose precipitated cardiac arrest. Resuscitation. 2021;159:69-76.

11. Bardai A, Lamberts RJ, Blom MT, Spanjaart AM, Berdowski J, van der Staal SR, et al. Epilepsy is a risk factor for sudden cardiac arrest in the general population. PloS one. 2012;7(8):e42749.

12. Stecker EC, Reinier K, Uy-Evanado A, Teodorescu C, Chugh H, Gunson K, et al. Relationship between seizure episode and sudden cardiac arrest in patients with epilepsy: a community-based study. Circulation Arrhythmia and electrophysiology. 2013;6(5):912-6.

13. Lamberts RJ, Blom MT, Wassenaar M, Bardai A, Leijten FS, de Haan G-J, et al. Sudden cardiac arrest in people with epilepsy in the community: Circumstances and risk factors. Neurology. 2015;85(3):212-8.

14. Legriel S, Bresson E, Deye N, Grimaldi D, Sauneuf B, Lesieur O, et al. Cardiac Arrest in Patients Managed for Convulsive Status Epilepticus: Characteristics, Predictors, and Outcome. Critical care medicine. 2018;46(8):e751-e60.

15. Eroglu TE, Folke F, Tan HL, Torp-Pedersen C, Gislason GH. Risk of out-of-hospital cardiac arrest in patients with epilepsy and users of antiepileptic drugs. British journal of clinical pharmacology. 2022;88(8):3709-15.

16. Youn CS, Choi SP, Yim HW, Park KN. Out-of-hospital cardiac arrest due to drowning: An Utstein Style report of 10 years of experience from St. Mary's Hospital. Resuscitation. 2009;80(7):778-83.

17. Grmec S, Strnad M, Podgorsek D. Comparison of the characteristics and outcome among patients suffering from out-of-hospital primary cardiac arrest and drowning victims in cardiac arrest. International Journal of Emergency Medicine. 2009;2(1):7-12.

18. Dyson K, Morgans A, Bray J, Matthews B, Smith K. Drowning related out-of-hospital cardiac arrests: characteristics and outcomes. Resuscitation. 2013;84(8):1114-8.

19. Claesson A, Druid H, Lindqvist J, Herlitz J. Cardiac disease and probable intent after drowning. The American journal of emergency medicine. 2013;31(7):1073-7.

20. Reynolds JC, Hartley T, Michiels EA, Quan L. Long-Term Survival After Drowning-Related Cardiac Arrest. The Journal of emergency medicine. 2019;57(2):129-39.

21. Ryan K, Bui MD, Johnson B, Eddens KS, Schmidt A, Ramos WD. Drowning in the United States: Patient and scene characteristics using the novel CARES drowning variables. Resuscitation. 2023;187:109788.

22. Reizine F, Michelet P, Delbove A, Rieul G, Bodenes L, Bouju P, et al. Development and validation of a clinico-biological score to predict outcomes in patients with drowning-associated cardiac arrest. The American Journal of Emergency Medicine. 2024;81:69-74.

23. Schober A, Sterz F, Handler C, Kurkciyan I, Laggner A, Roggla M, et al. Cardiac arrest due to accidental hypothermia--a 20 year review of a rare condition in an urban area. Resuscitation. 2014;85(6):749-56.

24. Legriel S, Bougouin W, Chocron R, Beganton F, Lamhaut L, Aissaoui N, et al. Early in-hospital management of cardiac arrest from neurological cause: Diagnostic pitfalls and treatment issues. Resuscitation. 2018;132:147-55.
