# Supplementary material for: The role of contributing factors, triggers, and prodromal symptoms in the etiological classification of out-of-hospital cardiac arrest; A scoping review
Source: PLoS One. 2025 Jul 16;20(7):e0327651. doi: 10.1371/journal.pone.0327651 (PMC12266415; doi:10.1371/journal.pone.0327651)
Supplement: S4 Appendix — (DOCX) [file pone.0327651.s004.docx]

**S 4 Appendix: Summary of included studies evaluating etiologies of exercise-related out-of-hospital cardiac arrest (OHCA)**

| **Author** | **Year/ Country** | **Study Design** | **Definition of**  **SR-OHCA occurrence** | **Source of initial etiologies data** | **Source of final etiologies data** | **Total population:**  **N** | **Initial etiologies** | **Final etiologies (if reported)** | **Triggers** |
| --- | --- | --- | --- | --- | --- | --- | --- | --- | --- |
| Berdowski, et al ^[^[^1^](#_ENREF_1)^]^ | 2013 Netherlands | Observational | During or within one hour of exercise | ARREST |  | 2,524 | Presumed cardiac |  | Exercise |
| Soholm, et al  ^[^[^2^](#_ENREF_2)^]^ | 2014  Denmark | Cohort | Within 15 min of cessation physical activity* | OHCA data  (Utstein style) | Hospital records | 1,393 | Presumed cardiac | Cardiac etiologies | Exercise |
| Edwards, et al ^[^[^3^](#_ENREF_3)^]^ | 2015  UK | Observational | During or within 1 hour of exercise** | LAS |  | 6,713 | Presumed cardiac |  | Exercise |
| Kiyohara, et al ^[^[^4^](#_ENREF_4)^]^ | 2017  Japan | Cohort | During exercise | OHCA registry  (Utstein style) |  | 16,278 | Presumed cardiac | 1-Cardiac diseases 2- Non-cardiac etiologies | Exercise |
| Viglino, et al ^[^[^5^](#_ENREF_5)^]^ | 2017  France | Observational |  | The Northern French Alp Cardiac arrest registry |  | 12,636 | Presumed cardiac | Cardiac diseases  Trauma  Respiratory  Others | Ski on slopes |
| Landry, et al ^[^[^6^](#_ENREF_6)^]^ | 2017  Canada | Observational | During, or within 1 hour after exertion of MET>3 during the activity | Epistry | Autopsy and medical reports | 74 | Presumed cardiac | 1-IHD  2-Primary arrhythmia 3-Hypertrophic cardiomyopathy  4- Structural cardiac disease  5-Unknown | Competitive and non-competitive exercise |
| Kiyohara, et al ^[^[^7^](#_ENREF_7)^]^ | 2017  Japan | Cohort | During exercise | OHCA registry (Utstein style) | Medical record | 11,059 | Presumed cardiac |  | Exercise |
| Ro, et al ^[^[^8^](#_ENREF_8)^]^ | 2017  South Korea | Observational | During exercise *** | EMS data |  | 6,273 | Presumed cardiac |  | Exercise and physical activity |
| Jung, et al ^[^[^9^](#_ENREF_9)^]^ | 2018  South  Korea | Observational | During exercise | Cardiovascular disease surveillance database | Hospital record | 1,835 | Presumed cardiac |  | Exercise in the mountain |
| Bohm, et al ^[^[^10^](#_ENREF_10)^]^ | 2020  Germany | Observational | During or within one hour of exercise | EMS data, FIFA, media | Autopsy | 349 | Presumed cardiac | Cardiac and  non-cardiac etiologies | Exercise and physical activities |
| Gerardin, et al ^[^[^11^](#_ENREF_11)^]^ | 2020  France | Observational | Before, during, and within 2 hours after end of race | Race-Paris data registries |  | 18 | Presumed cardiac | MI | Exercise and marathon running |
| Bohm et al ^[^[^12^](#_ENREF_12)^]^ | 2023  Germany | Observational | During or within one hour of cessation of competitive/recreational exercise | EMS reporting, (SDEC) registry; Confidential web-based data platform; the German Resuscitation Registry, | Adjudicated by 2 cardiologists and Autopsy report | 147 | Presumed cardiac | 1-CAD  2-Cardiomyopathy  3-Idiopathic VF  4-Aortic dissection | Exercise |
| Weizman, et al ^[^[^13^](#_ENREF_13)^]^ | 2023  Multicentre | Observational | During or within one hour of exercise | SDEC (Paris) ARREST, and SRCR |  | 760 | No obvious etiology | 1-Unknown  2-Idiopathic  3-MI  4-Cardiomyopathy  5-Electrical heart disease  6-Non-cardiac etiologies | Light-vigorous exercise activity |

**ARREST**: Amsterdam resuscitation studies. **CAD**: Coronary artery disease. **CPR**: Cardiopulmonary resuscitation. **EMS**: Emergency medical service. **FIFA**: Federation international football association. **IHD:** Ischemic heart disease **LAS**: London Ambulance Service**. MET**: Metabolic equivalent. **MI:** Myocardial ischemia. **OHCA:** Out-of-hospital cardiac arrest. **SDEC**: Sudden Death Expertise Center. **SRCR**: Swedish Register for Cardiopulmonary Resuscitation. **VF**: Ventricular fibrillation

*Exercise was defined as moderate to vigorous physical activity. Lawn mowing, housekeeping, and sexual intercourse were not considered as exercise.

** Engaging in at least moderate intensity exercise (running, swimming, cycling, or gym class) at the time or within 1 hour of arrest

*** Physical activity at the time of the incident was defined as one of following exercises: bicycling, conditioning exercises, dancing, fishing, hunting, sports, walking, running, water activities, and winter activities. OHCA occurring not during exercise defines as CA occurring during other activities, including home activity, inactivity, transportation, and occupation

**References**

1. Berdowski J, de Beus MF, Blom M, Bardai A, Bots ML, Doevendans PA, et al. Exercise-related out-of-hospital cardiac arrest in the general population: incidence and prognosis. European heart journal. 2013;34(47):3616-23.

2. Soholm H, Kjaergaard J, Thomsen JH, Bro-Jeppesen J, Lippert FK, Kober L, et al. Myocardial infarction is a frequent cause of exercise-related resuscitated out-of-hospital cardiac arrest in a general non-athletic population. Resuscitation. 2014;85(11):1612-8.

3. Edwards MJ, Fothergill RT. Exercise-related sudden cardiac arrest in London: incidence, survival and bystander response. Open heart. 2015;2(1):e000281.

4. Kiyohara K, Sado J, Matsuyama T, Nishiyama C, Kobayashi D, Kiguchi T, et al. Out-of-hospital cardiac arrests during exercise among urban inhabitants in Japan: Insights from a population-based registry of Osaka City. Resuscitation. 2017;117:14-7.

5. Viglino D, Maignan M, Michalon A, Turk J, Buse SK, Blancher M, et al. Survival of cardiac arrest patients on ski slopes: A 10-year analysis of the Northern French Alps Emergency Network. Resuscitation. 2017;119:43-7.

6. Landry CH, Allan KS, Connelly KA, Cunningham K, Morrison LJ, Dorian P, et al. Sudden Cardiac Arrest during Participation in Competitive Sports. The New England journal of medicine. 2017;377(20):1943-53.

7. Kiyohara K, Nishiyama C, Kiguchi T, Nishiuchi T, Hayashi Y, Iwami T, et al. Exercise-Related Out-of-Hospital Cardiac Arrest Among the General Population in the Era of Public-Access Defibrillation: A Population-Based Observation in Japan. Journal of the American Heart Association. 2017;6(6).

8. Ro YS, Shin SD, Song KJ, Hong KJ, Ahn KO. Association of Exercise and Metabolic Equivalent of Task (MET) Score with Survival Outcomes after Out-of-Hospital Cardiac Arrest of Young and Middle Age. Resuscitation. 2017;115:44-51.

9. Jung E, Park JH, Kong SY, Hong KJ, Ro YS, Song KJ, et al. Cardiac arrest while exercising on mountains in national or provincial parks: A national observational study from 2012 to 2015. The American journal of emergency medicine. 2018;36(8):1350-5.

10. Bohm P, Scharhag J, Egger F, Tischer K-H, Niederseer D, Schmied C, et al. Sports-Related Sudden Cardiac Arrest in Germany. The Canadian journal of cardiology. 2021;37(1):105-12.

11. Gerardin B, Guedeney P, Bellemain-Appaix A, Levasseur T, Mustafic H, Benamer H, et al. Life-threatening and major cardiac events during long-distance races: updates from the prospective RACE PARIS registry with a systematic review and meta-analysis. European journal of preventive cardiology. 2020:2047487320943001.

12. Bohm P, Meyer T, Narayanan K, Schindler M, Weizman O, Beganton F, et al. Sports-related sudden cardiac arrest in young adults. EP Europace. 2023;25(2):627-33.

13. Weizman O, Empana J-P, Blom M, Tan HL, Jonsson M, Narayanan K, et al. Incidence of cardiac arrest during sports among women in the European Union. Journal of the American College of Cardiology. 2023;81(11):1021-31.
