# Supplementary material for: The role of contributing factors, triggers, and prodromal symptoms in the etiological classification of out-of-hospital cardiac arrest; A scoping review
Source: PLoS One. 2025 Jul 16;20(7):e0327651. doi: 10.1371/journal.pone.0327651 (PMC12266415; doi:10.1371/journal.pone.0327651)
Supplement: S5 Appendix — (DOCX) [file pone.0327651.s005.docx]

**S 5 Appendix: Summary of included studies focused on the association of environmental factors with out-of-hospital cardiac arrest (OHCA) etiologies**

| **Author** | **Year/ Country** | **Design of study** | **Source of initial diagnosis data** | **Source of final diagnosis data *** | **Total population;**  **N** | **Initial etiology** | **Final etiology*** | **Triggers of OHCA** |
| --- | --- | --- | --- | --- | --- | --- | --- | --- |
| **Cold and hot ambient temperature** | | | | | | | | |
| Nishiyama, et al ^[^[^1^](#_ENREF_1)^]^ | 2011  Japan | Cohort | The Utstein Osaka registry |  | 10,723 | Presumed cardiac |  | Ambient temperature |
| Nakanishi, et al ^[^[^2^](#_ENREF_2)^]^ | 2011  Japan | Observational | AMI- Kyoto Multi-Centre Risk Study |  | 2,599 | Presumed cardiac |  | Ambient temperature |
| Tanigawa, et al ^[^[^3^](#_ENREF_3)^]^ | 2013  Japan | Cross sectional | EMS data |  | 48,911 | Presumed cardiac |  | Ambient temperature |
| Fukuda, et al ^[^[^4^](#_ENREF_4)^]^ | 2014  Japan | Observational | Utstein registry |  | 120,721 | Presumed cardiac | 1-Cardiac  2-Respiratory | Ambient temperature |
| Kang, et al ^[^[^5^](#_ENREF_5)^]^ | 2016  South  Korea | Time series  Case-crossover | Cardiovascular disease surveillance |  | 50,318 | Presumed cardiac |  | Ambient temperature |
| Yamazaki, et al ^[^[^6^](#_ENREF_6)^]^ | 2017  Japan | Time-stratified  case-crossover | Resuscitation registry |  | 97,500 | Presumed cardiac |  | Ambient temperature |
| Hensel, et al ^[^[^7^](#_ENREF_7)^]^ | 2018  Germany | Observational | Standardized electronic patient records |  | 2,445 | Presumed cardiac |  | Ambient temperature |
| Yoshinaga, et al ^[^[^8^](#_ENREF_8)^]^ | 2019  Japan | Case-crossover | EMS data (Utstein-style) |  | 1,452 | Presumed cardiac |  | Cold ambient temperature |
| Ryti, et al ^[^[^9^](#_ENREF_9)^]^ | 2022  Finland | Case-crossover | Helsinki CA Registry |  | 5,677 | Medical  (Utstein 2015) |  | Ambient temperature |
| Dai, et al ^[^[^10^](#_ENREF_10)^]^ | 2023  China | Observational | EAD |  | 12, 583 | Presumed cardiac |  | Cold ambient temperature |
| **Air Pollutants:** | | | | | | | | |
| Levy, et al ^[^[^11^](#_ENREF_11)^]^ | 2001  USA | Case-crossover | EMS reports | Autopsy report | 362 | Presumed cardiac | Confirmed cardiac | Air pollutant PM10  CO  SO_2_ |
| Forastiere, et al ^[^[^12^](#_ENREF_12)^]^ | 2005  Italy | Case-crossover | Regional OHCA registry |  | 5,144 | Presumed cardiac |  | Air pollution:  PNC  PM10  CO |
| Dennekamp, et al ^[^[^13^](#_ENREF_13)^]^ | 2010  Australia | case-crossover | VACAR (Utstein style) |  | 8,434 | Presumed cardiac |  | Air pollution: PM 2.5 |
| Silverman, et al ^[^[^14^](#_ENREF_14)^]^ | 2010  USA | Case-crossover  Time- series | New York OHCA database |  | 8,216 | Presumed cardiac |  | Air pollutant PM 2.5 |
| Rosental, et al ^[^[^15^](#_ENREF_15)^]^ | 2013  Finland | Case-crossover | EMS data  (Utstein style) | Autopsy and medical reports | 2,134 | Presumed cardiac | 1-AMI 2-Other cardiac etiologies | Air pollutant:  PM 2.5  O_3_ |
| Wichmann, et al ^[^[^16^](#_ENREF_16)^]^ | 2013  Denmark | Case-crossover | MECU  (Utstein style) |  | 4,657 | Presumed cardiac |  | PM 10  PM 10- 2.5 |
| Straney, et al ^[^[^17^](#_ENREF_17)^]^ | 2014  Australia | Case-crossover | St. John ambulance OHCA data |  | 8,551 | Presumed cardiac |  | Air pollution PM2.5  CO |
| Kang, et al ^[^[^18^](#_ENREF_18)^]^ | 2016  South  Korea | Case-crossover | Cardiovascular disease surveillance |  | 21,509 | Presumed cardiac |  | Air pollution PM2.5-10 |
| Ho, et al ^[^[^19^](#_ENREF_19)^]^ | 2018  Singapore | Time- stratified  case-crossover | PAROS |  | 8,589 | All etiologies | 1-Cardiac  2-Respiratory | Air pollutant: PSI |
| Kojima, et al ^[^[^20^](#_ENREF_20)^]^ | 2020  Japan | Case-crossover | Japan Utstein data registry |  | 103,189 | Presumed cardiac |  | Air pollutant:  PM 2.5 |
| Gentile, et al ^[^[^21^](#_ENREF_21)^]^ | 2021  Italy | Observational | OHCA registries (Utstein style) |  | 1,582 | Medical  (Utstein 2015) |  | Air pollutants: Benzen  PM 10  PM 2.5  CO |
| **Other environmental conditions:** | | | | | | | | |
| Dennekamp, et al ^[^[^22^](#_ENREF_22)^]^ | 2015  Australia | Case- crossover | VACAR  (Utstein style) |  | 2,046 | Presumed cardiac |  | Fire smoke; PM2.5 and CO |
| Andrew, et al ^[^[^23^](#_ENREF_23)^]^ | 2017  Australia | Time series | VACAR  (Utstein style) |  | 32 | Presumed cardiac | 1-Cardiac 2-Respiratory | Thunderstorm |
| Jones, et al ^[^[^24^](#_ENREF_24)^]^ | 2020  USA | Case-crossover | CARES |  | 5,336 | Presumed cardiac and  respiratory |  | Wildfire smoke  PM 2.5 |

**AMI**: Acute myocardial infarction. **CARES**: Cardiac arrest registry to enhance survival. **CO**: Carbon monoxide. **EAD**: Emergency ambulance dispatch. **MECU**: Mobile Emergency Care Unit. **EMS:** Emergency medical service. **OHCA**: Out-of-hospital cardiac arrest. **O_3_**: Ozone. **PAROS**: Pan-Asian Resuscitation Outcomes Study. **PM**: Particulate matter. **PNC**: Particle number concentration**. PSI**: Pollutant standards index. **SO_2_:** Sulfurous oxide**. VACAR**: Victorian ambulance cardiac arrest registries.

*Final etiologies and source of final etiologies if reported

**References**

1. Nishiyama C, Iwami T, Nichol G, Kitamura T, Hiraide A, Nishiuchi T, et al. Association of out-of-hospital cardiac arrest with prior activity and ambient temperature. Resuscitation. 2011;82(8):1008-12.

2. Nakanishi N, Nishizawa S, Kitamura Y, Nakamura T, Matsumuro A, Sawada T, et al. Circadian, weekly, and seasonal mortality variations in out-of-hospital cardiac arrest in Japan: analysis from AMI-Kyoto Multicenter Risk Study database. The American journal of emergency medicine. 2011;29(9):1037-43.

3. Tanigawa-Sugihara K, Iwami T, Nishiyama C, Kitamura T, Goto M, Ando M, et al. Association between atmospheric conditions and occurrence of out-of-hospital cardiac arrest- 10-year population-based survey in Osaka. Circulation journal : official journal of the Japanese Circulation Society. 2013;77(8):2073-8.

4. Fukuda T, Ohashi N, Doi K, Matsubara T, Kitsuta Y, Nakajima S, et al. Impact of seasonal temperature environment on the neurologic prognosis of out-of-hospital cardiac arrest: a nationwide, population-based cohort study. Journal of critical care. 2014;29(5):840-7.

5. Kang S-H, Oh I-Y, Heo J, Lee H, Kim J, Lim W-H, et al. Heat, heat waves, and out-of-hospital cardiac arrest. International journal of cardiology. 2016;221:232-7.

6. Yamazaki S, Michikawa T. Association between high and low ambient temperature and out-of-hospital cardiac arrest with cardiac etiology in Japan: a case-crossover study. Environmental health and preventive medicine. 2017;22(1):60.

7. Hensel M, Geppert D, Kersten JF, Stuhr M, Lorenz J, Wirtz S, et al. Association between Weather-Related Factors and Cardiac Arrest of Presumed Cardiac Etiology: A Prospective Observational Study Based on Out-of-Hospital Care Data. Prehospital emergency care : official journal of the National Association of EMS Physicians and the National Association of State EMS Directors. 2018;22(3):345-52.

8. Yoshinaga T, Shiba N, Kunitomo R, Hasegawa N, Suzuki M, Sekiguchi C, et al. Risk of Out-of-Hospital Cardiac Arrest in Aged Individuals in Relation to Cold Ambient Temperature - A Report From North Tochigi Experience. Circulation journal : official journal of the Japanese Circulation Society. 2019;84(1):69-75.

9. Ryti NRI, Nurmi J, Salo A, Antikainen H, Kuisma M, Jaakkola JJK. Cold Weather and Cardiac Arrest in 4 Seasons: Helsinki, Finland, 1997-2018. American journal of public health. 2022;112(1):107-15.

10. Dai M, Chen S, Huang S, Hu J, Jingesi M, Chen Z, et al. Increased emergency cases for out-of-hospital cardiac arrest due to cold spells in Shenzhen, China. Environmental science and pollution research international. 2023;30(1):1774-84.

11. Levy D, Sheppard L, Checkoway H, Kaufman J, Lumley T, Koenig J, et al. A case-crossover analysis of particulate matter air pollution and out-of-hospital primary cardiac arrest. Epidemiology (Cambridge, Mass). 2001;12(2):193-9.

12. Forastiere F, Stafoggia M, Picciotto S, Bellander T, D'Ippoliti D, Lanki T, et al. A case-crossover analysis of out-of-hospital coronary deaths and air pollution in Rome, Italy. American journal of respiratory and critical care medicine. 2005;172(12):1549-55.

13. Dennekamp M, Akram M, Abramson MJ, Tonkin A, Sim MR, Fridman M, et al. Outdoor air pollution as a trigger for out-of-hospital cardiac arrests. Epidemiology (Cambridge, Mass). 2010;21(4):494-500.

14. Silverman RA, Ito K, Freese J, Kaufman BJ, De Claro D, Braun J, et al. Association of ambient fine particles with out-of-hospital cardiac arrests in New York City. American journal of epidemiology. 2010;172(8):917-23.

15. Rosenthal FS, Kuisma M, Lanki T, Hussein T, Boyd J, Halonen JI, et al. Association of ozone and particulate air pollution with out-of-hospital cardiac arrest in Helsinki, Finland: Evidence for two different etiologies. Journal of Exposure Science & Environmental Epidemiology. 2013;23(3):281-8.

16. Wichmann J, Folke F, Torp-Pedersen C, Lippert F, Ketzel M, Ellermann T, et al. Out-of-hospital cardiac arrests and outdoor air pollution exposure in Copenhagen, Denmark. PloS one. 2013;8(1):e53684.

17. Straney L, Finn J, Dennekamp M, Bremner A, Tonkin A, Jacobs I. Evaluating the impact of air pollution on the incidence of out-of-hospital cardiac arrest in the Perth Metropolitan Region: 2000-2010. Journal of epidemiology and community health. 2014;68(1):6-12.

18. Kang S-H, Heo J, Oh I-Y, Kim J, Lim W-H, Cho Y, et al. Ambient air pollution and out-of-hospital cardiac arrest. International journal of cardiology. 2016;203:1086-92.

19. Ho AFW, Wah W, Earnest A, Ng YY, Xie Z, Shahidah N, et al. Health impacts of the Southeast Asian haze problem - A time-stratified case crossover study of the relationship between ambient air pollution and sudden cardiac deaths in Singapore. International journal of cardiology. 2018;271:352-8.

20. Kojima S, Michikawa T, Matsui K, Ogawa H, Yamazaki S, Nitta H, et al. Association of Fine Particulate Matter Exposure With Bystander-Witnessed Out-of-Hospital Cardiac Arrest of Cardiac Origin in Japan. JAMA network open. 2020;3(4):e203043.

21. Gentile FR, Primi R, Baldi E, Compagnoni S, Mare C, Contri E, et al. Out-of-hospital cardiac arrest and ambient air pollution: A dose-effect relationship and an association with OHCA incidence. PloS one. 2021;16(8):e0256526.

22. Dennekamp M, Straney LD, Erbas B, Abramson MJ, Keywood M, Smith K, et al. Forest Fire Smoke Exposures and Out-of-Hospital Cardiac Arrests in Melbourne, Australia: A Case-Crossover Study. Environmental health perspectives. 2015;123(10):959-64.

23. Andrew E, Nehme Z, Bernard S, Abramson MJ, Newbigin E, Piper B, et al. Stormy weather: a retrospective analysis of demand for emergency medical services during epidemic thunderstorm asthma. BMJ (Clinical research ed). 2017;359:j5636.

24. Jones CG, Rappold AG, Vargo J, Cascio WE, Kharrazi M, McNally B, et al. Out-of-Hospital Cardiac Arrests and Wildfire-Related Particulate Matter During 2015-2017 California Wildfires. J Am Heart Assoc. 2020;9(8):e014125.
