# Supplementary material for: The role of contributing factors, triggers, and prodromal symptoms in the etiological classification of out-of-hospital cardiac arrest; A scoping review
Source: PLoS One. 2025 Jul 16;20(7):e0327651. doi: 10.1371/journal.pone.0327651 (PMC12266415; doi:10.1371/journal.pone.0327651)
Supplement: S6 Appendix — (DOCX) [file pone.0327651.s006.docx]

**S 6 Appendix: Summary of included studies evaluating the association of prodromal symptoms with out-of-hospital cardiac arrest (OHCA) etiologies**

| **Author** | **year/**  **country** | **Study design** | **Source of initial etiology data** | **Source of final etiology**  **Data, if reported** | **Total population:**  **N** | **Initial etiology** | **Final etiology, if reported** | **Reported Prodromal Symptoms; n(%)** |
| --- | --- | --- | --- | --- | --- | --- | --- | --- |
| Kurckiyan, et al ^[^[^1^](#_ENREF_1)^]^ | 2000  Austria | Observational | Emergency medicine | Autopsy | 60 | PE |  | Dyspnea:41(68%) Syncope:29(48%) CP:15(25%) |
| Inamasu, et al ^[^[^2^](#_ENREF_2)^]^ | 2011  Japan | Cohort | EMS data registry | Medical chart | 250 | Presumed cardiac | SAH PN PE AA/AD | LOC:138 (55.2%) CP:70 (28%) |
| Nishiyama, et al ^[^[^3^](#_ENREF_3)^]^ | 2013  Japan | Cohort | Osaka OHCA data registry |  | 1,466 | Presumed cardiac  Presumed non-cardiac | **Cardiac etiologies**  **Non-cardiac etiologies:**  1-Cerebrovascular disease  2-Respiratory disease  3-Aortic disease  4-Malignancy | **Cardiac etiologies**  Prodromal symptom: 61% (p<0.003) CP Dyspnea Syncope:  **Non-cardiac etiologies**  Prodromal symptoms:70%  CP  Dyspnea  Syncope |
| Hoglung, et al  ^[^[^4^](#_ENREF_4)^]^ | 2014  Sweden | Case-crossover | MONICA myocardial registry | Hospital record | 403 | Presumed cardiac | MI | Health care consumption and prodromal symptoms:  **One week before OHCA**  CP:14%, p<0.001  GI symptoms :7.7%  Dyspnea :6.9  **Control Week:**  CP: 0  GI symptoms:1.2%  Dyspnea: 0.2% |
| Nehme, et al ^[^[^5^](#_ENREF_5)^]^ | 2015  Australia | Observational | VACAR  Utstein style |  | 1,056 | Presumed cardiac | not noted | CP:48.8% Dyspnea: 41.8% Altered consciousness: 37.8% |
| Arnaout, et al ^[^[^6^](#_ENREF_6)^]^ | 2015  France | observational | OHCA data Utstein style | ICU data | 258 | Neurological and non-neurological etiologies | 1-SAH  2-Subdural hematoma  3-Ischemic stroke  4-Intracerebral hematoma 5-Cardiac diseases 6-Respiratory | **Neurological symptoms:**  Headache  Impaired consciousness  Seizure  Neurological deficit  **Other prodromal symptoms:** CP  Dyspnea  Syncope |
| Marijon, et al ^[^[^7^](#_ENREF_7)^]^ | 2017  USA | Observational | EMS database | In-hospital and medical examiner | 839 | Presumed cardiac |  | CP  Dyspnea  Syncope/ palpitation  Others |
| Lee, et al ^[^[^8^](#_ENREF_8)^]^ | 2020  South Korea | Observational | EMS Data  Utstein style |  | 12,969 | 1-Cardiac  2-Non-cardiac 3-Other |  | No prodromal symptom:59.5% Neurological symptom: 14.2%^ Respiratory Symptoms: 12.5% Cardiac Symptoms :5% GI symptoms :5% **Cardiac etiologies with prodromal symptom**:37.6% **Non-cardiac etiologies with symptom**:48% |
| Nazerian, et al ^[^[^9^](#_ENREF_9)^]^ | 2022  USA | Observational |  | Medical chart | 280 |  | **ABI** Cerebral hemorrhage Spinal cord injury Ischemic stroke Status epilepticus  **Cardiac disease** ACS Cardiogenic shock **Hypoxemia** Pneumonia COPD | **OHCA due to ABI**:  NS^*^  CP  Dyspnea   **OHCA due to other etiologies:**  NS ^*^: n=6(2.3%), p<0.01  CP: n=65 (25%), p<0.01  Dyspnea: n=50 (19%), p:0.02 |

**AAD**: Ascending aortic dissection. **AA:** Aortic aneurysm. **ABI**: Acute brain injury. **ACS**: Acute coronary syndrome. **COPD**: Chronic obstructive pulmonary disease. **CP**: Chest pain. **EMS**: Emergency medical service.**GI**: Gastrointestinal. **ICU**: intensive care unit. **LOC**: Loss of consciousness. **MI**: Myocardial infarction. **NS**: Neurological symptoms. **OHCA**: Out-of-hospital cardiac arrest. **PE**: Pulmonary embolism. **PN:** Pneumonitis. **SAH**: Subarachnoid hemorrhage. **VACAR:** Victorian ambulance cardiac arrest registry

^ Neurological symptoms include mental change, seizure, headache, convulsion, dizziness, and paralysis

*Neurological symptoms: seizure, headache, and focal signs

**References**

1. Kurkciyan I, Meron G, Sterz F, Janata K, Domanovits H, Holzer M, et al. Pulmonary embolism as a cause of cardiac arrest: presentation and outcome. Archives of internal medicine. 2000;160(10):1529-35.

2. Inamasu J, Miyatake S, Tomioka H, Shirai T, Ishiyama M, Komagamine J, et al. Prognostic significance of acute pain preceding out-of-hospital cardiac arrest. Emergency medicine journal : EMJ. 2011;28(7):613-7.

3. Nishiyama C, Iwami T, Kawamura T, Kitamura T, Tanigawa K, Sakai T, et al. Prodromal symptoms of out-of-hospital cardiac arrests: a report from a large-scale population-based cohort study. Resuscitation. 2013;84(5):558-63.

4. Hoglund H, Jansson J-H, Forslund A-S, Lundblad D. Prodromal symptoms and health care consumption prior to out-of-hospital cardiac arrest in patients without previously known ischaemic heart disease. Resuscitation. 2014;85(7):864-8.

5. Nehme Z, Andrew E, Bray J, Cameron P, Bernard S, Meredith I, et al. The significance of pre-arrest factors in out-of-hospital cardiac arrests witnessed by emergency medical services: A report from the Victorian Ambulance Cardiac Arrest Registry. EMA - Emergency Medicine Australasia. 2015;27(SUPPL. 1):19.

6. Arnaout M, Mongardon N, Deye N, Legriel S, Dumas F, Sauneuf B, et al. Out-of-hospital cardiac arrest from brain cause: epidemiology, clinical features, and outcome in a multicenter cohort*. Critical care medicine. 2015;43(2):453-60.

7. Uy-Evanado A, Reinier K, Rusinaru C, Chugh H, Stecker EC, Jui J, et al. Warning symptoms and survival from sudden cardiac arrest. Circulation. 2018;138(Supplement 1).

8. Lee SY, Song KJ, Shin SD, Hong KJ. Epidemiology and outcome of emergency medical service witnessed out-of-hospital-cardiac arrest by prodromal symptom: Nationwide observational study. Resuscitation. 2020;150:50-9.

9. Nazerian P, De Stefano G, Lumini E, Fucini P, Nencioni A, Paladini B, et al. Comparison of out of hospital cardiac arrest due to acute brain injury vs other causes. The American journal of emergency medicine. 2022;51:304-7.
