# Supplementary material for: The role of contributing factors, triggers, and prodromal symptoms in the etiological classification of out-of-hospital cardiac arrest; A scoping review
Source: PLoS One. 2025 Jul 16;20(7):e0327651. doi: 10.1371/journal.pone.0327651 (PMC12266415; doi:10.1371/journal.pone.0327651)
Supplement: Attachment — Submitted filename: PONE-D-24-59166, Rebuttal Letter-Jan-2025.docx [file pone.0327651.s007.docx]

**Rebuttal Letter to address the editor’s concern (PONE-D-24-59166)**

Thank you to the editor for taking time to review and provide comments to improve the quality of this manuscript. This letter has been prepared to address the concerns that were raised following previous submission in Dec 2024.

| **Editor’s comments** | **The authors’ responses** |
| --- | --- |
| PLOS ONE considers systematic reviews and meta-analyses for publication; however, these submissions must address a clear and defined research question; conduct a systematic and comprehensive literature review; and use clearly reported, reproducible, and systematic methods to identify, select, and critically appraise relevant research. Our author guidelines for systematic reviews are at <https://journals.plos.org/plosone/s/submission-guidelines#loc-systematic-reviews-and-meta-analyses>.  In this case, we note that you have not specified whether both study selection and data extraction are performed in duplicate. We feel that this is necessary to minimize bias, and without this step we have concerns about the validity of the data included in the review and the conclusions that can be drawn | This is a scoping review not a systematic review.  We applied the scoping review methodology by following Arksey and O’ Malley’s methodological steps. The method section (pages# 5-8) explains the 6 steps of conducting this review. Prisma flow chart (Fig 1) has been prepared to demonstrate the process of selecting articles. Preferred Reporting Items for Systematic reviews and Meta-Analyses extension for Scoping Reviews (PRISMA-ScR) Checklist (S1 Appendix) has been used to report this scoping review.  For this review, the first author (SS) reviewed articles (titles, abstracts, and full texts) and abstracted data, and the senior author (LJM) verified selected articles and data abstraction [1-4]. Having the second reviewer to screen all citations was not possible as the volume to screen was excessive with over 20,000 titles. It could have been done by splitting the titles into a number of small batches and using a large number of reviewers with content expertise. The downside with this approach was the potential compromise of consistency in approach and the lack of enough individuals in the graduate school faculty with content expertise. This was a graduate student thesis and as such the supervisor role of intense oversight at each step was required. We felt methodologically for a scoping review that this was sufficient. Source of evidence selection (page# 7) explains the steps that were taken to minimize potential bias. Infeasibility of having the 2^nd^ reviewer was also reported as a limitation of this study (page# 39). |

**References:**

1.	Tricco AC, Lillie E, Zarin W, O’Brien K, Colquhoun H, Kastner M, et al. A scoping review on the conduct and reporting of scoping reviews. BMC Medical Research Methodology. 2016;16(1).

2.	Mak S, Thomas A. Steps for Conducting a Scoping Review. Journal of Graduate Medical Education. 2022;14(5):565-7.

3.	Arksey H, O'Malley L. Scoping studies: towards a methodological framework. International Journal of Social Research Methodology. 2005;8(1):19-32.

4.	Shaeri S, Considine J, Dainty KN, Olasveengen TM, Morrison LJ. Accuracy of etiological classification of out-of-hospital cardiac arrest: A scoping review. Resuscitation. 2024;198C:110199.
