# Supplementary material for: The role of contributing factors, triggers, and prodromal symptoms in the etiological classification of out-of-hospital cardiac arrest; A scoping review
Source: PLoS One. 2025 Jul 16;20(7):e0327651. doi: 10.1371/journal.pone.0327651 (PMC12266415; doi:10.1371/journal.pone.0327651)
Supplement: Attachment — Submitted filename: PONE-D-25-01818 Response to reviewers.docx [file pone.0327651.s008.docx]

**Response Letter to address the reviewers’ comments (PONE-D-25-01818)**

Thank you to the editor and reviewers for taking time to provide this thoughtful review and comments to improve the quality of this manuscript. All comments have been embedded in the manuscript with appropriate changes to make tracking these comments easier for reviewers. The authors have summarised the changes in the table below.

| **Journal requirements and reviewers’ comments** | **The authors’ responses** |
| --- | --- |
| **Journal requirements** |  |
| Thank you for stating the following financial disclosure:   [The primary author received 2019-2020 Graduate student funding from Institute of Health Policy, Management, and Evaluation and Graduate Student Endowment Funding from faculty of medicine, University of Toronto.].  Please state what role the funders took in the study.  If the funders had no role, please state: ""The funders had no role in study design, data collection and analysis, decision to publish, or preparation of the manuscript.""  If this statement is not correct you must amend it as needed.  Please include this amended Role of Funder statement in your cover letter; we will change the online submission form on your behalf.  25-May 2025  1. "We note that your Data Availability Statement is currently as follows: [All relevant data are within the manuscript and its Supporting Information files.]  Please confirm at this time whether or not your submission contains all raw data required to replicate the results of your study. Authors must share the “minimal data set” for their submission. PLOS defines the minimal data set to consist of the data required to replicate all study findings reported in the article, as well as related metadata and methods (https://journals.plos.org/plosone/s/data-availability#loc-minimal-data-set-definition).  For example, authors should submit the following data: - The values behind the means, standard deviations and other measures reported; - The values used to build graphs; - The points extracted from images for analysis. | We declare that “The funders had no role in study design, data collection and analysis, decision to publish, or preparation of this manuscript”  This is a scoping review to map current published articles. No meta-analysis has been conducted. Minimal data set of this scoping review, including a summary of included studies is available within the manuscript and S3-6 Appendices with relevant citations. The result of descriptive analysis is available in Table 2 (number and percentage). No mean and standard deviation have been measured. There is no graph in this submission. No points have been extracted from images for analysis. |
| **Reviewer #1** |  |
| The manuscript is organized, methodologically sound, and relevant to emergency medicine and cardiac arrest classification.  Overall Assessment of the Manuscript:  This manuscript presents a well-organized and methodologically robust scoping review of the factors, triggers, and early symptoms contributing to the etiological classification of out-of-hospital cardiac arrest (OHCA). The research is timely and essential, addressing a vital gap in understanding OHCA etiology, with significant implications for clinical practice and future research. Below is a comprehensive critique that evaluates the content, clarity, methodology, and adherence to PRISMA-ScR guidelines.Areas for Improvement  1. Justification for Scoping Review Approach (Minor Issue) Though the study utilizes a scoping review methodology, the rationale for selecting this approach could be articulated more effectively. Incorporate a brief statement in the introduction that clarifies why a scoping review (as opposed to a systematic review) was the most suitable choice  2. Transparency in Protocol Registration (Moderate Issue)  The manuscript notes that the protocol is registered with the Open Science Framework (OSF) but does not include a registration number or direct link. Add the OSF link for increased transparency.  3. Missing Detailed Search Strategy (Moderate Issue)  The search strategy is summarized but lacks comprehensive detail, such as Boolean operators, MeSH terms, and gray literature inclusion. Please Provide a complete search strategy in an accompanying appendix.  4. Justification for Excluding Critical Appraisal (Moderate Issue)  The manuscript does not explain why a formal quality assessment is excluded. Although PRISMA-ScR does not require such an assessment, a brief justification is essential. please Include a one-sentence rationale in the methods section.  5. Limitations Section Needs Expansion (Major Issue)  The limitations section is brief and does not discuss potential biases (e.g., selection bias, exclusion of non-English studies, reliance on EMS data). Please expand the limitations to include:◦ Potential selection bias due to language restrictions.◦ Inherent limitations of EMS data (e.g., misclassification of OHCA etiology).◦ Absence of a formal quality appraisal as a potential limitation.◦ Future research is necessary to validate findings with prospective cohort studies. | Thanks for comments, suggestions, and encouragement  1-The justification for scoping review is included in the introduction (Line# 85-87). We selected the scoping review because it was anticipated studies with diverse methodology (mostly observational or reflective) and limited number of publications. The purpose of this review was to map current evidence to identify the contributing factors, triggers, and prodromal symptoms of OHCA that may contribute to etiological classification of cardiac arrest. Since no systematic or scoping review has been conducted before, the scoping review was deemed to be the best option to map evidence and identify scientific evidence to address the study’s objective.  2-The OSF provides DOI, which is specific to this scoping review and directly links to the registration (Line # 100 and S1 Appendix). The registration number OSF.IO/K5ZDP was added to line #99.  3-Search strategy is available in S2 Appendix, including the chosen mesh terms and Boolean to search all three electronic databases, including Medline, Embase, and EBM-Cochrane databases.  We didn’t search gray literature, and this was noted as limitation of this scoping review (line#401). According to scoping review methodology, searching gray literature is not mandatory to conduct a scoping review [1]. We strongly preferred to map scientific evidence from published peer-reviewed studies for this scoping review to provide a rational knowledge synthesis and a firm foundation upon which to justify further research.  4-Critical appraisal is not mandatory for conducting a scoping review because the purpose of scoping review is to provide a comprehensive evidence map or an overview of current evidence regardless of the quality of evidence [1-3]. This was explained and cited in Line # 142. One sentence has been added to the limitation. (line#416-417)  5-We excluded the non-English literature according to our protocol and defined eligibility criteria (Table 1; Page#7) due to difficulty of translation or having someone with relevant expertise to translate the scientific text in foreign languages. This was already noted as the limitation of this study (Line# 401-402) and slightly revised.  We added one sentence to the limitation (line # 415-416) pertaining to the potential etiological misclassification of included studies. The purpose of this scoping review was not to evaluate the etiological misclassification. A prior scoping review highlighted a three-time discrepancy between initial etiological classification of OHCA based on EMS documentation using Utstein etiological classification and confirmed etiologies documented in medical charts or autopsy reports [4].This current scoping review was conducted to understand the role of contributing factors, triggers, and prodromal symptoms, which may help refine the etiological classifications. The finding of this review may strengthen the finding of previous scoping review on the potential etiological misclassification.  We included studies with observational, case-crossover, and time series that provide scientific evidence regarding the contributing factors, triggers, and prodromal symptoms of OHCA. The design of selected studies is available in S3-S6 Appendices. Future research would be suggested to integrate findings of this scoping review with real-life data. (Line#424) |
| **Reviewer #2** |  |
| Thank you for the opportunity to review this well-conducted scoping review.This is a systematic review aimed at identifying additional contributing factors, triggers, and symptoms of out-of-hospital cardiac arrest (OHCA) to supplement the existing Utstein criteria with further classification parameters. Overall, this systematic review has been conducted with sound scientific methodology.  However, it appears that a substantial number of studies have already examined the relationship between COVID-19 and OHCA. I find it difficult to understand why COVID-19 is considered such a significant influencing factor in OHCA, and I would appreciate clarification from the authors on this matter. The included studies in this systematic review seem to be heavily focused on COVID-19, which I would like to comment on and critically question.  Apart from this, the paper is coherently written, and this work represents a valuable contribution to the scientific understanding of OHCA and the development of more suitable quick alerts for identifying its etiology. | Thank you for your supportive comments.  Observational studies of COVID-19 pandemic and etiological classification of OHCA were targeted in the search as an example of a circumstance or contributing factor affecting the etiological classification of OHCA. The covid-19 pandemic as a contributing factor increased the likelihood of respiratory or medical cause to OHCA. One sentence has been added to clarify this section. (Line# 177) |

References:

1.	Tricco AC, Lillie E, Zarin W, O’Brien K, Colquhoun H, Kastner M, et al. A scoping review on the conduct and reporting of scoping reviews. BMC Medical Research Methodology. 2016;16(1).

2.	Peters MDJ, Marnie C, Tricco AC, Pollock D, Munn Z, Alexander L, et al. Updated methodological guidance for the conduct of scoping reviews. JBI Evidence Synthesis. 2020;18(10).

3.	Peters MD, Godfrey CM, Khalil H, McInerney P, Parker D, Soares CB. Guidance for conducting systematic scoping reviews. JBI Evidence Implementation. 2015;13(3):141-6.

4.	Shaeri S, Considine J, Dainty KN, Olasveengen TM, Morrison LJ. Accuracy of etiological classification of out-of-hospital cardiac arrest: A scoping review. Resuscitation. 2024;198C:110199.
